# Learning management system and e-learning tools: an experience of medical students' usage and expectations

**DOI:** 10.5116/ijme.57a5.f0f5

**Published:** 2016-08-20

**Authors:** David A. Back, Florian Behringer, Nicole Haberstroh, Jan P. Ehlers, Kai Sostmann, Harm Peters

**Affiliations:** 1 Bundeswehr Hospital Berlin, Department of Orthopedics and Traumatology, Germany; 2 Dieter Scheffner Center for Medical Teaching and Educational Research, Charité - Universitätsmedizin Berlin, Germany; 3 Charité Center Tumor Medicine, Department of Radiation Oncology and Radiotherapy, Charité - Universitätsmedizin Berlin, Germany; 4 Didactics and Educational Research in Health Science, University Witten-Herdecke, Germany

**Keywords:** Medical teaching, medical students, learning management system, e-learning tools, evaluation

## Abstract

**Objectives:**

To investigate medical students´ utilization of and problems with a learning management system and its e-learning tools as well as their expectations on future developments.

**Methods:**

A single-center online survey has been carried out to investigate medical students´ (n = 505) usage and perception concerning the learning management system Blackboard, and provided e-learning tools. Data were collected with a standardized questionnaire consisting of 70 items and analyzed by quantitative and qualitative methods.

**Results:**

The participants valued lecture notes (73.7%) and Wikipedia (74%) as their most important online sources for knowledge acquisition. Missing integration of e-learning into teaching was seen as the major pitfall (58.7%). The learning management system was mostly used for study information (68.3%), preparation of exams (63.3%) and lessons (54.5%). Clarity (98.3%), teaching-related contexts (92.5%) and easy use of e-learning offers (92.5%) were rated highest. Interactivity was most important in free-text comments (n = 123).

**Conclusions:**

It is desired that contents of a learning management system support an efficient learning. Interactivity of tools and their conceptual integration into face-to-face teaching are important for students. The learning management system was especially important for organizational purposes and the provision of learning materials. Teachers should be aware that free online sources such as Wikipedia enjoy a high approval as source of knowledge acquisition. This study provides an empirical basis for medical schools and teachers to improve their offerings in the field of digital learning for their students.

## Introduction

The goal of medical educational curricula is to provide students with knowledge and the clinical competence to treat patients at the best state-of-the-art; wherever possible, in an evidence-based manner.^1^ Up to the end of the last century, medical undergraduate education consisted mainly of face-to-face teachings, such as lectures, or self-directed use of paper-based books or lecture notes.^2^ With the rise of the internet, the former options have been expanded by a new mediation of knowledge via digital online-supported teaching and learning scenarios (e-learning) and thereby become part of most modern medical undergraduate curricula.^3^^-^^5^ The use of online-provided learning features and tools with its 24/7 availability and the possibility to offer more features than textbooks is very popular among students.^3^^-^^5^ E-learning has become a self-evident offering in modern medical faculties,^6^ and it is also compulsory implemented into medical curricula.^7^ Some e-learning scenarios provide uni-directional information for online use or downloads (e.g. lecture notes, presentations etc.),^5^ whereas others integrate highly interactive tools like radiological diagnosis quizzes or virtual patients.^8^^-^^10^ Also the use of so-called Web 2.0 media with podcasts, wikis or blogs has been discovered for medical teaching.^11^^,^^12^ However, most presented offers are limited to single projects or have focused only on individual medical disciplines,^5^^,^^9^^,^^13^ with only sparsely organized approaches on any medical school level.^14^

Nowadays, the provision of e-learning tools or complex blended learning scenarios depends on an online platform where students and teachers can get access to them.^4^ To address this issue, different forms of learning management systems (LMS) were established in medical faculties worldwide. A LMS can be defined as software that automates the administration, tracking, and reporting of training events and delivers learning contents rapidly.^15^ It also enables the provision of a structured curriculum, and eases both the accessibility of many contents and an all-in-one organization.^16^ Many studies have already reported on the use of LMS in undergraduate and even postgraduate medical teaching for the provision of e-learning contents or the access to libraries or discussion forums.^10^^,^^13^^,^^16^^-^^18^ Also complex teaching concepts as spiral curricula are supportable by LMS.^19^

A sufficient familiarity of teachers with electronic media and LMS has to be regarded as the basis of modern teaching.^20^^,^^21^ In order to ease the compliance of students to use digital teaching amendments, it is highly important that medical teachers know about potential pitfalls and students’ experiences, wishes or needs regarding the conception, design or provision of e-learning scenarios, tools and LMS. ^22^ However, until today, only few studies have concentrated on individual disciplines or topics instead of LMS as the main focus^13^^,^^23^ – and those few who did were again linked to certain disciplines rather than to a medical school level. Also students´ problems with the use of e-learning tools are relevant, but only rarely reported in medical fields.^24^ Overall, there is a lack in comprehensive empirical data on medical students´ use of digital learning tools and learning management systems as well as their expectations on the provision of those offerings.

The undergraduate curriculum at German medical schools consists of 12 semesters, characterized by a differing mix of lectures, seminars, bedside teachings, practical trainings, internships and problem-based learning. While LMS are implemented at almost all medical schools, e-Learning offerings are mostly voluntary and embedded into blended learning concepts.^25^ The purpose of this study was to examine medical students’ needs and expectations concerning e-learning tools and their learning management system, based on their current utilization.

## Methods

### Study design

The current study was designed as a single-center online survey and has been carried out at the Charité – Universitätsmedizin Berlin, Germany. The department of e-learning informed Medical students about the purpose of the study. 

### Participants

Medical students from all semesters were included in the study, as long as they were enrolled for medical undergraduate studies at the Charité. Participation was voluntary and anonymous. Formal and ethical approval was obtained from the institutional data protection office and ethic committee (Ethikkommission, Ethikaussschuss 1 am Campus Charité-Mitte; No. EA1/081/12).

### Sample size and sampling procedures

Participants were asked for participation via email announcements. In the period from June to September 2012, 505 students voluntarily responded to the email announcement and followed an included link to the survey provided online. The response rate was 11%.

### Data collection methods

The aim of the questionnaire was to investigate the sample's usage and perception concerning a learning management system and its e-learning tools, as well as their expectations on future developments.

The learning management system used was Blackboard Academic Suite with the components Learning-, Community- und Content-System (© Blackboard Inc., Washington DC, USA). The system is accessible for all registered students of the medical school via regular internet access and their use of an individual password. Furthermore, all students have a password-protected internet access to the medical school´s online library and hold an institutional, separate email account.

Lecture notes or scripts were provided mostly as PowerPoint (Microsoft PowerPoint, Microsoft Corp., USA) and PDF (Adobe System Inc., USA) scripts. Podcasts were produced with Camtasia Studio (Version 5.1.0, TechSmith Corp., USA). Virtual patient cases were provided by the CAMPUS authoring system (Version 1.3.2827 © 2006, University of Heidelberg, Germany). Discussion forums or quiz formats were part of the LMS Blackboard features. The wiki system “WikiBlog” was based on the Team collaboration software Confluence (Atlassian, Australia) and the blogs by the open source web software WordPress MU (Free Software Foundation, USA).

This online survey could not be complemented with data of tracking and recording of students´ LMS using behavior due to legal data security regulations in Germany. In order to assess medical student’s perceptions, needs and expectations when using the LMS or e-learning tools, a standardized questionnaire was developed which was consistent with prevalent quality criteria of internet surveys. ^26^^-^^28^ Based on a PubMed and MEDLINE search and exploratory interviews with professional experts, the focused subject was structured and questions were developed. The preliminary questionnaire was validated by employing a pre-test method to narrow down the final questions.^29^ To analyze content validity, the adapted questionnaire was sent to a panel of independent experts in the field of educational research and e-learning within the faculty. Following a peer review process, the final questionnaire was generated, and it composed 70 questions. Items were then categorized into 2 domains, the first domain which covered medical student’s prevailing use of the LMS or e-learning tools, and the second domain which comprised their needs and expectations (concerning the LMS or e-learning tools).

The item sets of the first domain were related to online sources used by students for the acquisition of knowledge (11 items), online sources used by medical students for teamwork with other students (11 items), e-learning tools used by medical students for their studies (11 items), and the purpose of using a learning management system (8 items).

The item sets of the second domain were related to perceived difficulties when using the learning management systems and e-learning tools (9 items), key characteristics of online learning platforms and e-learning tools for the learning process (13 items), and students’ needs concerning the online learning platforms and e-learning tools provided by the medical school Charité (7 items).

Participants were either asked to indicate their agreement with each item on a five-point Likert scale, with 1 indicating ‘strongly disagree’ and 5 ‘strongly agree’, or assess their usage on a five point intensity scale by 1 indicating ‘never’ and 5 ‘very frequently’. All items had a “non-applicable” option.

Additionally, students were asked to narratively answer the question, how – according to their own personal wishes or expectations – the ideal learning management system and its e-learning offerings should be designed.

### Data collection procedure

The survey was completed online via Survey Monkey (SurveyMonkey Inc., Oregon, USA). Respondents had the opportunity to change their answers by using a back button until they were ready for a final submission of the survey. Their responses were first documented anonymously in the confidential database of Survey Monkey and then transferred to a local secure server.

### Data analysis

Descriptive data were collected, analyzed and reported as mean plus/minus standard deviation or as relative frequency in relation to the total numbers. Free-text answers were analyzed for repetitive sequences by two independent reviewers of the faculty. Qualitative research was conducted in order to obtain more information about students´ perception of e-learning offerings. A systematic rule guided qualitative text analysis was applied using techniques of qualitative content analysis according to Mayring.^30^ All analyses were performed using SPSS statistical software.

## Results

Within the three-month duration of the study, 505 students participated in the survey (of 4629 addressed). The mean age was 24.2 years (SD 4.4) and the mean study semester was 6.5 (SD 3.8). Of all participants, 340 (67.3%) were female and 161 (31.9%) were male. 4 students did not indicate their gender.

### Students´ current use of LMS and e-learning tools

The most popular online sources for the acquisition of knowledge, which were used “very often” or “often”, were Wikipedia (74%) and lecture notes provided by the medical school faculty (73.7%), followed by medical online portals (57.8%). Among the least-used online sources for learning were YouTube (9%), social networks (6.8%), and iTunes U as podcast portal (5.1%). 

For the online exchange and teamwork with other students on study issues, students used most preferably emails (52.8% “often” or “very often”), followed by Facebook (32%) and online storage providers (17.3%). One’s own webpages or blogs (85.4% “never”) or professional network portals as Xing (98.7% “never”) or LinkedIn (98.8% “never”) were used least.

The ranking of the most popular tools used in e-learning was led by lecture slides (77.7%), videos (71.9%) and digital texts (71.3%). The lowest ranked tools were simulations (33.4%), serious games (13.8%) and discussion forums (7%).

When asked what purpose they used the LMS for, the students´ leading reasons were to gain organizational study information (68.3%), for the preparation of exams (63.3%) and as preparation and post-processing of lessons (54.5%). On the other hand, the importance level of the LMS for communicating with other students (2.2%) or teachers (1.9%) or keeping lists or calendars (1.3%) was vanishingly low. During the semester, 38.6% of all students used the LMS daily, 48.3% on a weekly basis, and 13.1% less than once a week.

### Needs assessment concerning LMS and e-learning tools

In respect to the leading problems seen with e-learning concepts, LMS and e-learning tools, participants complained about missing integration of contents into lessons by teachers (58.7%), poor structure of the offers (57.8%), problems in actually locating these (56.9%), and a lack of interactivity (56.2%). Further comments considering problems with e-learning activities focused on missing personal contact (38.1%), too much additional learning effort (25.7%), or technical problems (22.6%).

When asked about the most important key characteristics of LMS and online tools which support their learning, the students ranked the “Top 5” with clarity (98.3%), teaching-related contents (92.7%), ease of use (92.5%), practice-orientation (91%) and time-saving (85.2%). A facilitation of collaboration with other students e.g. within forums (37.7%) or the provision of online tutors (33.2%) was regarded as least important by the participants.

**Table 1:** Students’ opinions on how e-learning should be designed* (N = 323)

Topic	Description of the desired contents
Interactive knowledge tests (n = 123)	Possibility for training and self-tests, for applying and checking knowledge (e.g. interactive exercises, quizzes, multiple-choice questions, training tests) preferably with direct feedback in any case of wrong answers
Completeness (n = 97)	Availability of all relevant contents (lecture notes, podcasts/videos), links and information for an intensive preparation and post-processing. Better access to e-books, marking of exam-relevant contents and add-ons
Clear structure (n = 65)	Uniform and clear structure of the teaching contents offered, e.g. ordered by semester or topics with emphasis on actual contents with concurrent possibility to access older contents
Relevance for learning objectives (n = 55)	Special marking and weighting of exam-relevant topics and online accessibility of all exam-relevant information
Practical relevance (n = 47)	Contents with relevance for practical clinical work, to understand theoretical knowledge or global coherences (e.g. operation videos). Realistic case examples to apply and deepen the gained knowledge
Contingency and actuality (n = 40)	More consistency of the offers (some modules have an extensive e-learning offer, versus others without any offer at all). Specific and always up-to-date offer for all courses. Technical and didactic training of the teachers. Uniform layout for all courses to enable a better clarity
Multimedia (n = 37)	Lecture notes/text files for pooling of knowledge, but e.g. practical videos in case of complex coherences. Additional scoring/visualization of lectures. Application of media features which are specific for the particular contents while considering the individual type of learning
Technical user-friendliness (n = 32)	Cutback on technical operation barriers (e.g. additional software, plug-ins etc.). Compatibility for all operating systems. Unrestricted mobile access via tablet or smartphone.
Easy access (n = 27)	No access barriers (different passwords, plug-ins, etc.), but one central and easily accessible offer (especially one uniform password)
Contact/commentary features (n = 23)	Integrated and uncomplicated possibility for contacts between students, but also with teachers. Possibility to communicate via a forum about certain contents.
Efficient learning (n = 23)	Compact provision of relevant information, which allows an efficient and time-saving gain and immersion of knowledge
Data transfer (n = 9)	Possibility to transfer internal teaching contents to private hard drives / to save or to print them for learning offline

*Multiple answers were allowed.

Among the needs concerning LMS and e-learning tools, which students had wished they received in the medical school, top-ranked were a unified online study portal (75.9% “strongly agree” or “agree”) and enough online memory (56.6%) or online portfolio systems (43.8%), while wikis (38.3%) or blogs (31.8%) were required less often. Only 24.1% shared the opinion that there was no need for any improvement as there were already enough alternatives available online.

Qualitative comments to the question, as to how the ideal LMS and its e-learning offerings should be designed, were analyzed using techniques of qualitative content analysis. An inductive classification of categories was conducted resulting in a summarizing content analysis with topics shown in Table 1 (n = 323).

## Discussion

Provision of e-learning elements via learning management systems (LMS) is one of the key factors for a successful introduction and both effective and persistent usage of digitally supported learning concepts in medical education. ^4^^,^^7^ This single-center survey enlightened some aspects of interest in a comprehensive approach concentrating on students´ use of a LMS and its e-learning tools as well as assessing their needs concerning those offerings on a medical school level. To our knowledge it is the first approach to address those topics in medicine without being connected to any single specific course or discipline.^13^ Concerning online knowledge sources in general, the medical school´s own teaching offers such as lecture notes were well liked, but were placed as being used at almost the same level as Wikipedia – far ahead of e.g. professional online journals. Also in other studies, Wikipedia was considered to be a relevant source of knowledge by students.^31^^,^^32^ Despite such encouraging data,^33^ there are also reasonable doubts among professionals concerning the actual reliability of Wikipedia’s contents.^34^^,^^35^ The results presented here underline the necessity to foster studies about students’ media competencies and their learning behaviors. However, the findings also seem to recommend e.g. for national associations of individual medical disciplines that one should check articles in Wikipedia which deal with their area of expertise, and to correct or even edit them in a proven and certified manner. Future studies will have to address the question how students´ use of such internet sources can be positively influenced by offering instructive and well-approved digital learning tools.

As a central headline of students´ preferences for the LMS and its e-learning contents, the term “efficacy” could be stated. Offers should be clearly structured, easy to operate, time saving, praxis-oriented and focused on curricular lessons. Additionally, a good communication about where to find certain offers seems to be needed. Interestingly, fun with any offers or an especially attractive layout was seen by the majority of participants to be less important. This might be quite important for clinical teachers who will mostly be rather short of time 36 and could thus invest more effort on the provision of fact-oriented contents than on a sophisticated design.^37^ At the same time it can be stated that most students regarded only few e-learning tools to be relevant for study preparation. Knowledge was apparently expected to be taught in as compact a manner as possible, resulting in a preference of those tools which seemed to lead quickly to a learning success, e.g. texts or videos rather than discussion forums or wiki-blogs. However, such a view on the results has to be seen critically, as still at least one-third of the participants supported forums and online tutoring. Along with this, there are examples of successful embedding among students using tools as discussion forums.^13^^,^^38^^,^^39^ Others showed that their local populations appreciated discussion forums most, whereby downloadable data were only seen to be present in the midfield and a use of emails even at the end.^13^ It can be postulated that the ranking of different tools within a LMS will always depend on an individual course’s structure – e.g. when a close tutor contact is ensured and there is a steady encouragement of using the tools.^38^

Regarding the above-mentioned students´ needs and concluded demands for teachers, faculty development approaches to support teachers´ familiarity with the LMS used and its established e-learning tools and services do seem to be recommendable.^37^ When asking for students´ priorities of electronic tools provided by the medical school, a uniform study portal was regarded as most important. This can be based on the practical log-in use and easy handling of e-learning materials; students would then not have to switch between individual platforms. This issue was also supported by the free-text answers, where students requested an easy access to the LMS and its contents. Thus, it seems to be recommendable that medical schools offer one single LMS to offer all digital learning components.^13^ The “number one” purpose for the LMS usage was the acquisition of information about curricular contents, followed by learning for exams and the preparation and post-processing of seminars. Communication tools of the LMS were used less, whereas students’ answers had indicated that communication itself was a relevant aspect.  In this context, it was interesting that emails were still the main line of communication in the evaluated population. That could be explained together with a well-established email service of the university. Just as other authors have already suggested an embedding of libraries into LMS,^40^ there could be a potential in connecting universities´ email accounts of students with a firmly established LMS into one password-protected platform.^13^

Students´ statements showed a strong desire for an active involvement in learning. Thus, e-learning offerings should include the possibility of interaction to promote the acquisition of knowledge.^9^ However, this aspect should be discussed critically. On the one hand, new features of the Web 2.0 and other means of teaching might be suitable to attract students´ attention,^12^^,^^39^ and these are also demanded by some authors.^41^ This might also be a new and promising approach for the population evaluated here, whereby Facebook was not apparently considered to be especially important as source of learning, but rather secondary concerning online communication about study-related aspects. On the other hand, it should also be taken into careful consideration that concentrating on proven tools might be more effective for a medical school and its teachers in aiming to provide a good and satisfying education than to follow too much of any current hype.^37^ Another interesting aspect taken from the free-text answers was the wish for retention of access to earlier learning materials. This could be important in the context of evidence-based medicine approaches with spiral learning concepts,^19^^,^^42^ where a LMS could connect different learning levels by linking related contents.

Some limitations have to be recognized for this study. As the answers had to be given online, this could have led to a non-response bias, attracting only participants interested in the internet.^43^ In addition, this was a single-center survey, and that limits its generalizability. There might be a bias that it was mostly those things addressed or answered which were also represented at our medical school. To learn more about this issue, this survey should be repeated in a multi-center approach on a national or international level. Either way, students´ answers will be influenced by the experiences they made at their medical school only. As that could give additional objective information about the using behavior of tools provided in a LMS, analyses of students´ behavior and preferences when using the LMS should be included and addressed in future studies.

## Conclusions

By providing organizational information and learning materials, a LMS can serve as a decisive component of a medical school to support the use of e-learning materials. In our study, the leading purpose to use the LMS was the acquisition of information about curricular contents, followed by learning for exams and the preparation and post-processing of seminars. Students wanted the LMS to support an efficient learning, with clear, practice-oriented contents, which are easy to use. Thus, we conclude that teachers should put more effort on the provision of fact-oriented contents than on a sophisticated design. However, interactivity and integration into face-to-face teaching are important aspects in the perception of e-learning tools and should be supported. Teachers should be aware that free online sources as Wikipedia enjoy a high approval as source of knowledge acquisition. As this is a serious issue for medical education, national associations of individual medical disciplines should consider editing online texts in an approved manner. Finally, students´ demand for remaining access to earlier learning materials could support the use of LMS and e-learning tools in the context of spiral learning concepts. The data presented here could serve as an empirical basis for medical schools to improve their offers in the field of digital learning for students.

### Acknowledgements

The authors thank Mrs. Tina Harms for her participation in the preparation of the evaluation questionnaire and data analysis.

### Conflict of Interest

The authors declare that they have no conflict of interest.
